# Hematolymphoid neoplasms involving the breast: A single institution clinicopathologic study of 59 patients

**DOI:** 10.1007/s00277-026-06901-9

**Published:** 2026-04-10

**Authors:** Jasmine Vickery, Rayne Peerenboom, Faiza Siddiqui, Jeeva Alphonsa Joy, Swetha S. Prabu, Daniel A. Arber, Sandeep Gurbuxani, Anna Biernacka, Girish Venkataraman

**Affiliations:** 1 Department of Pathology, University of Chicago, 5841 S Maryland Ave, Chicago, IL 60637 USA; 2 Cedars-Sinai, 8700 Beverly Blouvard, Los Angeles, CA 90048 USA; 3 Department of Pathology, Hospital of the University of Pennsylvania, 3400 Spruce Street, Philadelphia, PA 19104 USA; 4 Government Kilpauk Medical College, Chennai, 600010 Tamil Nadu India

**Keywords:** Breast, Lymphoma, Primary breast lymphoma, Secondary breast lymphoma, Extranodal lymphoma, Hematolymphoid neoplasm, Hematopoietic neoplasm

## Abstract

Breast hematopoietic neoplasms (BHN) are rare and may either primarily (PBHN) or secondarily (SBHN) involve breast tissue. The widespread use of needle biopsy for diagnosis of breast lesions necessitates knowledge of their presentation, radiologic aspects, histomorphology, and outcomes for seeking appropriate hematopathology consultation. Herein, we present our clinical experience as an academic institution and referral center of BHN in the past 20 years. We identified 59 patients diagnosed at the University of Chicago Medical Center between 2002-2021. Demographic, pathologic, radiologic, therapy, relapse data, and vital status were abstracted. Data were examined using univariable statistics with event-free and overall survival (EFS, OS) as primary outcomes examined with the lymphoma subgroup using Cox PH regression adjusted for age. The cases included 27 (46%) PBHN and 32 (54%) SBHN in a cohort comprising 93% females, mostly white (56%). The mean age at diagnosis was 58.8 years. Lymphomas were the most frequent BHN (86.4% of all cases). Patients with primary breast lymphomas (PBL) were significantly older than those with secondary breast lymphomas (SBL) (61.2 vs. 49.8 yrs, p<0.02). The most frequent lymphomas were extranodal marginal zone lymphoma (MZL) (32.2%) and diffuse large B-cell lymphoma/high grade B-cell lymphoma, not otherwise specified (DLBCL/HGBCL, NOS) (33.9%). Over half of MZLs and DLBCLs were primary in the breast. Within B-cell lymphomas, 20 (37%) were high grade with inferior 10-yr overall survival (OS) (age-adjusted HR 5.47, 95% CI 1.38, 21.64) compared to low-grade without any impact on event free survival (EFS). This is one of the largest cohorts so far describing BHN. DLBCL and MZL remain the most common lymphomas involving this site. Most patients were diagnosed via core needle biopsy (CNB) and did not have a prior BHN. Radiographically, the presentation may closely mimic breast carcinoma. FDG PET is a mainstay in the diagnosis and management of BHN.

## Introduction

Breast hematopoietic neoplasms (BHN) comprise a group of lesions that are morphologically and biologically heterogeneous [1]. They may occur as a “primary” disease originating within mammary tissue or as a “secondary” phenomenon by a preceding extramammary neoplasm, concurrent widespread systemic disease at the time of diagnosis, or disease recurrence. Lymphoma is the most common non-breast solid tumor to metastasize to the breast [2, 3]. The overall prevalence of lymphoma among breast cancers is low. A study found out of 19,354 breast malignancies; 11 patients were affected by primary breast lymphoma (0.06% of breast malignancies) and 23 cases of secondary breast lymphoma (0.12% of breast malignancies) [4]. Conversely, breast lymphoid neoplasms (BLN) account for < 5% of extranodal lymphomas and < 1% of non-Hodgkins’s lymphomas [5]. Despite the overall paucity their recognition is imperative for patient management. Several BHN may closely morphologically replicate the much more common epithelial breast neoplasms. The cytologic features of a high-grade lymphoid neoplasm appearing like a poorly differentiated carcinoma is a well-documented diagnostic pitfall [6]. Retrospective review of amended pathology reports for breast surgical specimens at a large academic medical center (during a 5-year period) found lymphoma mistaken for invasive carcinoma was 1 of 14 major diagnostic changes [7]. Core needle biopsies (CNB) are now the standard procedure for initial diagnosis of all breast lesions and diagnosis of a BHN can be reliably made on a CNB in most cases [6]. A series assessing diagnostic accuracy of breast CNB found an accurate definitive diagnosis was provided in 86% of cases, an “atypical” but not definitive diagnosis in 11% of cases, and 1 misdiagnosis as carcinoma (3%) [6]. When faced with this differential it is critical to be as accurate as possible as the rendered final diagnosis may drastically change clinical management. Some BHN do not need invasive surgery and if misdiagnosed as a breast carcinoma, patients with a BHN may undergo unnecessary surgery (in the breast and/or axilla). If breast carcinoma with a dense lymphoid infiltrate is misclassified as benign or misdiagnosed as a BHN this may delay the primary treatment which is surgery. BHN remain challenging lesions due to relatively small numbers, lack of pathologist experience, potentially limited amount of material on a CNB, and broad differential diagnosis.

Before describing our findings designation as primary versus secondary breast involvement by BHN, particularly lymphomas, has been somewhat controversial. Most studies have restricted the definition of primary tumors to those limited to the breast (one or both breasts) at diagnosis [8]. Many authors included synchronous ipsilateral axillary lymph node involvement to be designated as a primary lymphoma if breast tissue was the first clinical presentation and in the absence of systemic disease [8]. Traditionally, older studies have used diagnostic criteria that included cases in which the breast was the first or major site of presentation as primary tumors, even if on subsequent staging investigations the lymphomatous process was shown to involve distant nodal sites (Ann Arbor Stage III) or bone marrow (Ann Arbor Stage IV) [9]. Many authors have used the distinction between primary breast lymphoma (PBL) and secondary breast lymphoma (SBL) based on criteria first defined by Wiseman and Liao in 1972 [10]. In our study we use this same criteria, a PBL is a lymphoid neoplasm which at the time of the diagnosis clinically the breast was considered the site of primary or major manifestation of the lymphoma, is present exclusively at the level of the breast, with or without ipsilateral involvement of the axillary lymph nodes (only patients categorized at Ann-Arbor’s stages IE or IIE) and without a prior history of lymphoma of a similar histologic type in other areas of the body. The remaining lymphomas are categorized as secondary. Studies including this one by restricting the use of primary tumors to those limited to the breast and excluding cases that have disseminated disease at diagnosis may underestimate the actual incidence of BHN originating in the breast. We undertook this study to investigate the epidemiologic, clinicopathologic, and radiologic characteristics with the full spectrum of BHN.

## Methods

Study Design: After obtaining approval from the University of Chicago Medical Center Institutional Review Board, our health system’s pathology database was retrospectively reviewed from January 1, 2002, to July 31, 2021 for hematopoietic neoplasms involving the breast that were biopsied or excised during the course of their disease. Patients that were either referred to our institution for treatment or their slides were reviewed in consultation were included. For patients diagnosed within this period demographic, pathologic, radiologic, therapy, relapse/follow-up information, and vital status were abstracted from clinical, radiology, and pathology notes.

Patient characteristics and Data Collection: For this cohort we reviewed pertinent patient demographics age, sex, and medical history of breast carcinoma or autoimmune disease. Clinical and pathologic characteristics including specimen type for initial diagnosis, gross size of breast lesion, site of breast involvement, histologic type, initial diagnostic site whether it was nodal, extranodal within the breast, or elsewhere were recorded. Morphologic review was performed for all cases and included immunohistochemical stains when available. Recurrence date, first relapse site, and recurrence histologic type were reviewed. Clinical information including whether the lesion was clinically palpable and presence or absence of an implant were also investigated. Type of initial treatment (i.e., radiotherapy, and/or chemotherapy) was collected. For a subset of patients with a history of epithelial breast cancer the histologic type, grade, day of diagnosis and the time interval to BHN involvement was assessed. Certain radiologic information if available was abstracted from mammography reports including presence or absence of calcifications, round or irregular margins, if the mass was described as circumscribed, spiculated, or ill-defined, and if the lesion was uni- or multifocal. If the patient had no mammographic abnormality but one was performed this was noted. A small subset of patients had available MRI and/or FDG-PET information. If a PET scan was performed, avidity was noted. Follow up information including date of last follow up, vital status, and date of death were reviewed.

Statistical Analysis and Study Endpoints: Data were examined using univariable descriptive statistics. For survival analysis, we evaluated event-free and overall survival (EFS, OS) with analysis time extending from date of breast involvement by lymphoma (not date of primary lymphoma in secondary cases) until progression/transformation (for EFS only) or death due to any cause (all-cause mortality) censoring patients alive at last follow up as primary outcomes examined with the lymphoma subgroup using Cox PH regression adjusted for age. Additional variables were not included in the analysis since the analysis would be underpowered given the number of total events in both OS and EFS analysis.

## Results

Demographics and Clinical Findings: A total of 59 cases were identified, 4 males and 55 females. Most patients were white (56%) (Table 1). The mean age at diagnosis was 58.8 years. The size of the dominant breast lesion ranged from 0.6 to 10.0 cm. The cases included 27 (46%) primary breast hematopoietic neoplasms (PBHN) and 32 (54%) secondary breast hematopoietic neoplasms (SBHN). Of the lymphomas, patients with primary breast lymphoma (PBL) were significantly older than those with secondary breast lymphoma (SBL) (61.2 vs. 49.8 yrs, *p* < 0.02). There was no tumor size difference between PBL vs. SBL (median 2.1 cm). Half of the patients in our cohort presented with a palpable mass (29, 49%). There was no significant side predominance (*p* > 0.05). Most patients 47 (80%) were diagnosed via core needle biopsy (CNB) as the only specimen type. For the remaining patients 3 had an ultrasound guided CNB, 3 excisional biopsy alone, 1 had both a CNB and excisional biopsy, 2 had both a fine needle aspiration (FNA) and CNB, 1 FNA alone, 1 lumpectomy, and 1 punch biopsy. The initial diagnostic site was breast for most patients (32, 54%). Other sites included 5 cervical lymph node only, 1 concurrent breast and cervical lymph node biopsy, 1 breast and ipsilateral axillary lymph node, 1 axillary lymph node only, 1 both breast and chest wall, 1 breast skin, 6 peripheral blood and/or bone marrow. Other diagnostic sites included orbit, parotid, and stomach. The diagnosis of a BHN was incidental for 38 patients (64%) and the breast was the site of relapse for 37 (63%). 27 patients had lymph nodes as other sites of involvement, 29 patients had extranodal disease outside of the breast, and 17 had both nodal and extranodal involvement elsewhere. The most common nodal site of involvement were the axillary lymph nodes (14 patients total, 13 unilateral, 1 bilateral). Of these, 5 patients had ipsilateral axillary lymph nodes as their only other site of involvement. The most common other extranodal sites involved were bone marrow (14, 24%), skin/dermis (6, 10%), and peripheral blood (5, 8%). Five patients were identified with both a history of breast lymphoma and breast carcinoma (BC) (Table 2). See Table 1 for information on laterality and imaging below in Radiology section.

**Table 1:** Comparison of demographic, clinical, radiologic, and pathologic characteristics between primary and secondary breast lymphomas

Characteristics	Total (59)	Primary (32)	Secondary (27)	*P*-value
Age at diagnosis, median (IQR)	59 (46–67)	60 (46–68)	48 (41–63)	*0.056*
Race				
White	33 (56)	17 (53)	16 (59)	*0.25*
Black or African American	7 (12)	5 (16)	2 (7)	
Latinx	2 (3)	0 (0)	2 (7)	
Unknown	17 (29)	10 (31)	7 (26)	
Sex, Female	55 (93)	30 (94)	25 (93)	*1.00*
Side				
Right	27 (46)	15 (47)	4 (15)	*0.061*
Left	27 (46)	17 (53)	10 (37)	
Bilateral	4 (7)	0 (0)	12 (44)	
Unknown	1 (2)	0 (0)	1 (4)	
Palpable				*0.28*
Yes	29 (49)	14 (44)	15 (56)	
No	17 (29)	11 (34)	6 (22)	
Unknown	13 (22)	7 (22)	6 (22)	
Other nodal site				
Present	27 (46)	9 (28)	18 (67)	***0.003***
Absent	32 (54)	23 (72)	9 (33)	
Other extra-nodal site				
Present	29 (49)	8 (25)	21 (78)	***< 0.001***
Absent	30 (51)	24 (75)	6 (22)	
Concurrent breast CA				
Present	5 (9)	3 (9)	2 (7)	*1.00*
Absent	54 (92)	29 (91)	25 (93)	
Incidental				***< 0.01***
Yes	38 (64)	16 (50)	4 (15)	
No	20 (34)	15 (47)	23 (85)	
Unknown	1 (2)	1 (3)	0 (0)	
Size, median (IQR)	2.2 (1.3–3.6)	2.2 (1-5.4)	2.1 (1.6–3.1)	*0.73*
Calcifications				*0.68*
Present	10 (17)	5 (16)	5 (19)	
Absent	19 (32)	11 (34)	8 (30)	
Unknown	30 (51)	16 (50)	14 (52)	
Radiographic shape				*0.15*
Round	13 (22)	9 (28)	4 (15)	
Irregular	15 (25)	6 (19)	9 (33)	
Unknown	31 (53)	17 (53)	14 (52)	
Margin				*0.44*
Circumscribed	10 (17)	7 (22)	3 (9)	
Ill-Defined	12 (20)	5 (16)	7 (22)	
Spiculated	6 (10)	3 (9)	3 (9)	
Unknown	31 (53)	17 (53)	14 (44)	
Focality				*0.099*
Multifocal	7 (12)	2 (6)	5 (19)	
Unifocal	24 (41)	16 (50)	8 (30)	
Unknown	28 (47)	14 (44)	14 (52)	
Histologic Type				--
MZL	19 (32)	13 (41)	6 (22)	
DLBCL	18 (30)	10 (31)	8 (30)	
FL	9 (15)	5 (16)	4 (15)	
CLL/SLLL	3 (5)	2 (6)	1 (4)	
HGBCL, NOS	2 (3)	1 (3)	1 (4)	
B-lymphoblastic	2 (3)	0 (0)	2 (7)	
Histiocytic Sarcoma	1 (2)	0 (0)	1 (4)	
PTCL, NOS	1 (2)	1 (3)	0 (0)	
T-lymphoblastic	1 (2)	0 (0)	1 (4)	
ALCL, ALK+	1 (2)	0 (0)	1 (4)	
Mycosis Fungoides	1 (2)	0 (0)	1 (4)	
Extramedullary myeloid	1 (2)	0 (0)	1 (4)	
EBV				*1.00*
Positive	7 (12)	4 (13)	5 (19)	
Negative	9 (15)	3 (9)	4 (15)	
Unknown	43 (73)	25 (78)	18 (67)	
Treatment				***0.023***
Chemo/radiation	47 (80)	22 (69)	25 (93)	
Untreated	12 (20)	10 (31)	2 (7)	

Percentages may not sum to 100 due to rounding. Numbers may not add up due to unsufficient data

*P-values* represent Chi-Square or Fisher’s exact for categorical variables (as appropriate for sample size), or Wilcoxon rank sum for continuous variables

**Table 2:** Clinical and pathologic characteristics of patients with both breast lymphoma and breast carcinoma

Patient number	Lymphoma				Carcinoma			
	Category	Side	Histology		Side	Histology	Timing	Outcome after BL
1	PBL	L	DLBCL		L	IDC and DCIS	Concurrent	Unknown
2	SBL	R	FL		R	DCIS	FL preceding	A, 197 months
3	SBL	L	CLL/SLL		L	IDC	CLL/SLL preceding	A, 122 months
4	PBL	L	FL		L	IDC	Breast carc preceding	A, 48 months
5	PBL	L	DLBCL		Bil.	IDC	Breast carc preceding	A, 18 months

Abbreviations: A, alive; Bil., bilateral; BL, breast lymphoma, carc, carcinoma; CLL/SLL, chronic lymphocytic leukemia/small lymphocytic leukemia; DCIS, ductal carcinoma in situ; DLBCL, diffuse large B-cell lymphoma; IDC, invasive ductal carcinoma; FL, follicular lymphoma, L, left; PBL, primary breast lymphoma; R, right; SBL; secondary breast lymphoma

Radiology: The radiologic features are summarized in Table 1. Calcifications were identified on mammogram associated with the breast lesion for 10 patients and absent for 32 patients. When available the radiographic shape was most described as irregular (25%) and ill-defined (20%). Most lesions were reported as unifocal (24 patients). One patient had a mammogram with no mammographic evidence of malignancy. Of note, 4 patients were reported to have PET scans and for all 4 the breast masses were PET-FDG avid by radiologic impression.

Pathology: Lymphomas were the most frequent BHN (Fig. 1). Examining the BLN the lymphoma cohort comprised 86.4% of all cases. The most frequent lymphomas were MZL (32.2%) and DLBCL/HGBCL, NOS (33.9%), followed by FL (15%) (Fig. 1) including two DLBCLs that transformed from a preceding FL. Over half of MZL and DLBCLs were primary in the breast with two MZL patients having underlying autoimmune disease. Although 2 patients had breast implants, no cases of breast implant-associated anaplastic large cell lymphoma (BIA-ALCL) were diagnosed. Interestingly, of the patients who had breast implants one was diagnosed with histiocytic sarcoma and was noted on MRI to have a unifocal hyperintense mass that was described as abutting the implant capsule. The other patient with implants was diagnosed with B-lymphoblastic lymphoma (B-ALL) and had a prior history of chronic myelogenous leukemia (CML). However, this patient’s disease was multifocal and bilateral it is unclear if these lesions were associated with or involved the implant capsule.

Therapy: Most patients (80%) underwent some therapeutic intervention and 11 patients were observed; for 13 patients the treatment was unknown. Most treated patients underwent chemotherapy alone (14, 24%), closely followed by chemotherapy and radiation together (11, 19%). None underwent radiation or surgery as the sole intervention. Three patients (5%) had surgery and chemoradiation, one patient (2%) had lumpectomy and radiation without chemotherapy. Of the six patients who had other therapies this included 1 patient with topical steroids alone, 1 patient with a history of BC and BLN had chemoradiation and lumpectomy for the carcinoma but observation only for the BLN (CLL/SLL). 1 patient had chemotherapy and IVIG, 1 patient had chemotherapy and CAR-T cell therapy, 1 had lumpectomy with short course steroids, and 1 chemoradiation as well as an allogenic stem cell transplant. Patients with high grade lymphomas including T-cell lymphoma who received chemotherapy all received CHOP-based regimens.

Outcomes: The patients with available follow-up information (53) most are alive (43, 81%) with follow-up time ranging from 18 to 197 months. 9 patients succumbed to their disease, most of the patients who died had DLBCL/HGBCL-NOS (5, 50%). The remaining deceased patients had histiocytic sarcoma (1), myeloid sarcoma (1), and T-lymphoblastic lymphoma (1). One patient with marginal zone lymphoma died of other causes. Within B-cell lymphomas, 20 (37%) were high grade with inferior 10-yr OS (age-adjusted HR 5.47, 95% CI 1.38, 21.64) compared to low-grade without any impact on EFS (Fig. 2). Two patients with an initial FL grade 1–2 transformed to DLBCL on relapse and that relapse was within the breast parenchyma. Another patient’s breast biopsy performed in 2008 showed FL grade “1–2/3” and subsequent left axillary lymph node biopsy in 2010 was diagnosed as “DLBCL”. There was one patient who had a prior history of T-cell large granular lymphocytic leukemia then DLBCL (non-germinal center subtype) diagnosed with a subsequent breast core needle biopsy.

## Discussion

BHN are often a diagnosis of exclusion. Distinguishing a low-grade B-cell neoplasm from benign chronic inflammatory infiltrates related to infection, foreign body/surgical site reactions, fat necrosis, lymphocytic (formerly known as diabetic) mastopathy, IgG4-sclerosing mastitis, cystic neutrophilic granulomatous mastitis, and idiopathic granulomatous mastitis can be problematic [5, 11]. Extranodal MZL is a low-grade B-cell lymphoma composed of small lymphoid cells that can have prominent plasmacytic differentiation. Of extranodal MZLs monoclonal plasma cells are most frequently found in cases involving the breast and the frequency of lymphoepithelial lesions in this site is the lowest [12]. Chronic inflammatory processes do not express monotypic immunoglobulin or show clonal rearrangement of IGH and are usually associated with a less dense lymphoid infiltrate and more fibrosis [13]. Immunohistochemistry may be helpful in this distinction as studies in the breast have shown that greater than 60% CD20 + B cells were present in 75% of MZL and only 23% of benign cases [13]. Greater than 40% CD3 + T-cells were exclusively seen in benign cases [13]. The t(11;18)(q21;q21) has been identified in the majority of breast MZL and may aid in the diagnosis [5].

Malignant BHN can be difficult to distinguish from breast carcinoma with abundant tumor infiltrating lymphocytes (TILS) otherwise previously known as “medullary carcinoma”. The rare entity lymphoepithelioma-like carcinoma of the breast can notoriously have such intense lymphocytic infiltration that it obscures the presence of the malignant epithelial component [14, 15]. Of particular diagnostic interest are few exceptional reported cases such as a follicular dendritic cell sarcoma with mucoid pools mimicking a primary mucinous carcinoma [16]. Additionally, the sarcomatoid variant of Anaplastic Large Cell Lymphoma (ALCL) presenting as a rapidly enlarging palpable breast lesion has been reported in a patient without breast implants [17]. DLBCL can closely mimic poorly differentiated breast carcinoma by presenting as solid nests, cords, and/or single epithelioid cells with moderate cytoplasm and vesicular nuclei. Leukemic involvement by AML may be particularly difficult to distinguish from invasive lobular carcinoma (ILC) because of the presence of relatively small, infiltrative neoplastic cells, single-file non-cohesive growth pattern, and lack of immunoreactivity for E-cadherin [18]. Both B and T-cell phenotypes of BLN can display frequent signet ring cells [19]. Immunohistochemistry for keratins such as AE1/3 can be vital for diagnosis. Lymphoid neoplasms will be negative for keratin. Although most BLN will have a triple negative immunohistochemical profile, low positivity for ERα can rarely be observed [6]. Up to 45% of DLBCL can show p63 expression [20]. Negative staining for keratins with p63 positivity can also be seen in metaplastic breast carcinomas and is not specific. BHN may not just mimic but occur in association with in-situ and invasive breast carcinomas. Co-existent metastatic carcinoma and lymphoma have been reported previously in axillary lymph nodes [21]. Even a “collision tumor” consisting of synchronous carcinoma with closely associated malignant lymphoma presenting as a single mass in the breast has been described [22].

Most BHN are lymphoid neoplasms. Our findings are similar to other reports that DLBCL is the most common histologic subtype followed by MZL and FL [1, 5]. BLN other than B-cell lineage including leukemic, myeloid, histiocytic, plasma cell, and T-cell neoplasms are rare, reported as case reports or small case series. Our findings reflect this as our cohort included single cases of extramedullary myeloid tumor, ALCL (ALK+), and histiocytic sarcoma (Fig. 3). Other uncommon but notable BHN include Burkitt lymphoma, plasmacytoma/plasma cell myeloma, myeloid sarcoma, Hodgkin’s Lymphoma, Rosai-Dorfman disease, ALK-positive histiocytosis of the breast, follicular dendritic cell sarcoma, interdigitating dendritic cell sarcoma, Hairy cell leukemia, and blastic plasmacytoid dendritic cell neoplasm [23–25]. Most PBL are of B-cell lineage, but T-cell malignancies such as extranodal NK/T cell lymphoma, T-cell lymphoblastic lymphoma, and subcutaneous panniculitis-like T-cell lymphoma have also been reported [26–29]. We did not observe any cases of BIA-ALCL, the most common BHN arising in association with breast implants [30]. Although histiocytic sarcomas have been described in the breast and axilla, there have been no other reported cases of a histiocytic sarcoma associated with a breast implant [31, 32]. At variance with some studies, we did not see aggressive lymphomas arising in women of childbearing age during pregnancy in our cohort [33–35].

It is important to consider all BHN as in up to 40% of patients a breast core-needle biopsy may be the first diagnosis of a hematologic malignancy [6]. In our study 38 of the 59 patients (64%) the diagnosis of BHN was incidental and not known prior to tissue sampling. Including a case of FL involving all lymph nodes of an axillary lymph node dissection some of which were also involved by metastatic carcinoma (Fig. 4). Most of our patients were diagnosed via CNB only. Excisional biopsy has been the traditional gold standard for the diagnosis of large B-cell lymphomas and in older series many patients with BLN underwent complete excision or mastectomy [36, 37]. It has been shown that mastectomy offers no benefit in recurrence risk or survival and subjects patients to the morbidity of a larger surgery [36]. Ultrasound guided core needle biopsy (US-CNB) has become the preferred method for the initial evaluation of breast lesions. Studies evaluating its diagnostic value in non-palpable suspicious breast lesions have shown high sensitivity, specificity, and diagnostic accuracy [38–40]. US-CNB for assessing lymphadenopathies suspected of lymphoma has also been found to be fast, effective, and safe with high diagnostic accuracy [41–42]. For superficial lymphadenopathies ultrasound elastography, which evaluates tissue stiffness, has emerged as a promising adjunct to US-CNB to improve diagnostic accuracy [43]. Excision should be reserved for patients where limited tissue sampling does not yield a definitive diagnosis or to remove refractory disease after treatment.

Like other studies we found overlapping imaging features of BLN with BC, making prospective clinical suspicion of breast lymphoma challenging [44]. We found that 17% of patients with a BHN including BLN in our study were described in radiology reports as associated with mammographic calcifications. This contrasts with prior studies of BLN in which calcifications are almost always absent [45, 46]. Interestingly, a case report of primary MZL in the breast has been documented presenting as grouped calcifications discovered during screening mammography [46]. This clinical presentation seems outstandingly rare.

Accurate and complete whole-body imaging is essential for staging and classification of BHN. FDG PET with CT of breast lymphoma demonstrates high glucose uptake and all four patients who had PET imaging in our study the breast masses were FDG PET avid by radiologic impression [47]. Although not specific, FDG PET-CT is incredibly useful and has a definitive role in every step of management of breast lymphomas including diagnosis, staging, treatment response, and detection of recurrence [47]. Occult involvement of the spleen and liver can be difficult to assess despite being an adverse prognostic factor. Accurate and sensitive assessment of spleen and liver by using fused FDG PET/contrast-enhanced CT scans can aid in characterization of subdiaphragmatic involvement [48]. Also, equivalence between PET/MRI and PET/diagnostic CT in properly defining disease stage has been shown in Hodgkins lymphoma and compared with FDG-PET/unenhanced MRI, staging with FDG-PET/full dose contrast-enhanced CT necessitates nonionic iodinate medium infusions and considerably higher amounts of ionizing radiation exposure [49]. For patients with estrogen receptor positive BC and BHN, PET/CT using 16α-[18 F]-fluoro-17β-estradiol (FES) may improve staging [50].

Treatment of BHN especially PBL has been somewhat contentious. Traditionally complete resection of a PBL was performed. Now multiple studies have shown no significant improvement in prognosis with upfront surgery which may delay systemic chemotherapy [51, 52]. In our study most patients were treated with chemotherapy alone. Treatment largely depends on the stage and BHN histologic subtype. Chemotherapy, especially the CHOP regimen, is recognized as the standard treatment for primary DLBCL [51]. Some low-grade B-cell subtypes of PBL such as FL and CLL/SLL can be observed without early intervention [52].

The occurrence of BLN and BC in the same patient has been reported to be relatively common. In a study 24% of patients with a BLN had either a history of or a concurrent BC [53]. We observed a much lower incidence, 5 of 59 patients (0.08%). It is interesting that most patients in our study developed BLN and BC in the same breast, likely due to small sample size. Some have asserted that the occurrence of BLN and BC in the same patient does not appear to have an adverse clinical significance in terms of prognosis [53]. Our data is too limited to draw any conclusions regarding prognosis due to heterogeneity in biology precluding a grouped cohort analysis.

The main limitation of this study is its retrospective nature. The true incidence of hematolymphoid lesions involving the breast is difficult to estimate, and many of the cases in this study were seen in consultation. In conclusion, although BHN are much less common than epithelial malignancies it is important to consider the full spectrum of hematopoietic tumors while viewing a breast core needle biopsy. US-CNB should be first line in the diagnostic work-up of lesions with clinical suspicion of lymphoma within the breast and/or lymph nodes. Low-grade lesions, especially B-cell lymphomas, can imitate benign reactive inflammatory infiltrates and high-grade neoplasms can appear virtually identical to invasive breast carcinoma. It is crucial to be aware of these entities for primary diagnosis and appropriate hematopathology consultation, even in patients without a prior history of hematologic malignancy.

### General abbreviations

Hematopoietic neoplasms involving breast (BHN), Primary breast hematopoietic neoplasms (PBHN), Secondary breast hematopoietic neoplasms (SBHN), Breast lymphoid neoplasms (BLN), Primary breast lymphoma (PBL), Secondary breast lymphoma (SBL), Breast carcinoma (BC), Invasive ductal carcinoma (IDC), Ductal carcinoma in situ (DCIS), Scarff-Bloom-Richardson (SBR), Tumor infiltrating lymphocytes (TILS), Invasive lobular carcinoma (ILC), Core Needle Biopsy (CNB), Ultrasound guided core needle biopsy (US-CNB), Fine Needle Aspiration (FNA), Hematoxylin and Eosin (H&E), Terminal duct lobular unit (TDLU), Event free survival (EFS), Overall survival (OS), Hazard ratio (HR), Fluorodeoxyglucose Positron Emission Tomography (FDG PET). 16α-[18 F]-fluoro-17β-estradiol (FES). See Fig. 1 for Hematopoietic neoplasm abbreviations.

### Hematopoietic neoplasm abbreviations

Non-Hodgkin Lymphoma (NHL), Marginal zone lymphoma (MZL), Extranodal marginal zone lymphoma of mucosa-associated lymphoid tissue (MALT), Diffuse large B-cell lymphoma (DLBCL), Follicular lymphoma (FL), Chronic lymphocytic/Small lymphocytic leukemia/lymphoma (CLL/SLL), High-grade B-cell Lymphoma Not Otherwise Specified (HGBCL, NOS), Peripheral T-cell lymphoma (PTCL), Extramedullary myeloid tumor/Acute myeloid lymphoma (AML), Breast implant associated Anaplastic Large Cell Lymphoma (BI-ALCL), Anaplastic Large Cell Lymphoma Anaplastic lymphoma kinase–positive (ALCL, ALK+), B-lymphoblastic lymphoma (B-ALL), Chronic myelogenous leukemia (CML).

### Ethics declaration

The institutional IRB at the University of Chicago Biological Sciences Division determined that the protocol is considered exempt because the retrospective research involves no more than minimal risk to the subjects, the research could not practicably be carried out without the requested waiver or alteration, the research does not use identifiable private information or identifiable biospecimens, the waiver or alteration will not adversely affect the rights and welfare of the subjects and whenever appropriate, the subjects or legally authorized representatives will be provided with additional pertinent information after participation. The work described has been carried out in accordance with the Declaration of Helsinki of the World Medical Association.

### Consent to participate

**Declaration**: This study received ethical approval from the institutional IRB at the University of Chicago Biological Sciences Division. This is an IRB-approved retrospective study, all patient information was de-identified and patient consent was not required. Patient data will not be shared with third parties.

### Consent to publish

**Declaration**: The authors affirm that human research participants provided informed consent for publication of the anonymized patient information to be published in this article including the images and information in Tables 1 and 2; Figs. 1, 2 and 3 (a-f), and 4. The participants have consented to the submission of the cases reported to the journal. Furthermore, the covered entity does not have actual knowledge that the information could be used alone or in combination with other information to identify an individual who is the subject of the information.

### Declaration of conflicting interests

The authors declare no potential conflicts of interest with respect to the research, authorship, and/or publication of this article.

## Data Availability

The data supporting the findings of this study are available within the article. The additional data is not publicly available due to containing clinical information that could compromise the privacy of research participants. Additional data is available on reasonable request from the corresponding author.
